# 

**Published:** 1887-12-24

**Authors:** 


### THE STUDENTS' COLUMN.

### UNIVERSITY OF LONDON.

### M.D. EXAMINATION.

Pass List.—Arkle, Charles Joseph, University College; Bahadhurgi, Kaikhosro Nasarvanji, B S., University College; Barnett, Lawrence, University College; Bright, Eustace Frederick, University College; Carr, John Walter, B.S., University College; Cocking, William Tusting,* University College; Davies, William Thomas Frederick, B.S., Guy's; Dingley, Arthur William, University College; Evans, Willmott Henderson, B.Sc., University College; Gow, William John,* St. Bartholomew's; Green, Charles David, B.S., St. Thomas's; Halstead, George Ezra, B.S., B.A., B.Sc., Guy's; Harsant, Joseph George, B.S., Guy's; Hichens, Frank, B.S., London; Hull, Walter, St. Thomas's; Jefferson, Arthur John, B.S., St. Thomas's; Joule, John Samuel, University College and St. Bartholomew's; Lewtas, John, St. Thomas's; Marsh, George Ryding, Guy's; Paley, Frederick John, St. Bartholomew's; Randell, Reginald Maurice Henry, Guy's; Rayne, Charles Alfred, B.S., University College; Robinson, Henry Betham, B.S., St. Thomas's; Swain, James, B.S., Westminster; Thomas, John Raglan, St. Barthomew's; Trevelyan, Edmond Fauriel, B.Sc., St. Bartholomew's; Turner, Philip Dymock (Gold Medal), University College; Washbourn, John Wychenford, B.S., Guy's; Wild, Robert Briggs,* Manchester Royal Infirmary and Owens College; Williamson, Richard Thomas, B.S., Owens College.

Logic and Psychology only.—Thomson, St. Clair, King's College.

* Obtained the number of marks qualifying for the Medal.

### EXAMINATION IN SUBJECTS RELATING TO PUBLIC HEALTH.

Pass List.—Collins, William Job, M.D., M.S., B.Sc. (Gold Medal), Private study; Pearce, Walter, M.D., B.S., B.Sc., St. Mary's; Smith, Kenneth Rawlings, M.D., B.S., University College.

### M.B. EXAMINATION.

PASS LIST.

First Division.—Balgarnie, Wilfred, St. Bartholomew's; Barendt, Frank Hugh, Liverpool Royal Infirmary; Barwise, Sidney, Queen's and Mason Colleges, Birmingham; Braddon, William Leonard, Guy's; Brook, William Henry Breffit, St. Bartholomew's; Brown, Herbert Henry, University College; Caldecot, Charles, Guy's; Calvert, John Telfer, St. Thomas's; Clarke, William Frederick, Guy's; Deanesly, Edward, B.Sc., University College; Featherstone, William Barltrop, Queen's College, Birmingham; Finley, Frederick Gault, McGill University, London Hospital, and Vienna; Flemming, Percy, University College; Gee, Frederick William, University College; Gill, Rainsford Foster, University College; Godfrey, Albert Edward, Northampton General Infirmary and St. Thomas's; Holder, Sydney Ernest, University College; Jecks, Cyril William, University College; Kelsall, Henry Truman, London Hospital; Kidd, Hugh Cameron, St. Thomas's; Lawrence, Thomas William Pelham University College; Lys, Henry Grabham, London Hospital; Marriott, Hyde, B.Sc., University College; Moynihan, Berkeley George Andrew, Yorkshire College; Nevins, John Ernest, Liverpool School of Medicine and Guy's; Rennie, George Edward, B.A. Sydney, University College; Risdon, William Newt, Guy's; Routh, Charles Frederic, Guy's; Smith, Hugh, London Hospital; Solly, Ernest, St. Thomas's; Stedman, Frederic Osmund, Charing Cross Hospital; Sunder, Charles Edward, University College; Taylor, Frederick Howard, London Hospital; Thane, Edgar Herbert, University College; Thompson, James Edwin, Owens College and Manchester Royal Infirmary; Tubby, Alfred Herbert, Guy's; Tunstall, John Ogle, University College; Vincent, Herbert Edmund, Guy's; Wheatley, James, King's College; Wheaton, Samuel Walton, St. Thomas's; Williams, William Griffith, St. Bartholomew's; Winter, Walter Essex, Middlesex and St. Bartholomew's.

Second Division.—Bailey, William Henry, St. Bartholomew's; Chapman, Harry Cecil, St. Bartholomew's; Clegg, Joseph, Owens College and Manchester Royal Infirmary; Colman, Walter Stacy, University of Edinburgh and University College; Crouch, Charles Percival, St. Bartholomew's; Fisher, Theodore, Guy's; Furnivall, Bryan, St. Bartholomew's; Gault, Arthur Henry, Owens College and Manchester Royal Infirmary; Heatherley, Francis, Guy's; Jordan, Walter Ross, Birmingham Medical School; Luff, Arthur Pearson, B.Sc., St. Mary's; Mumford, Alfred Alexander, Owens College and Manchester Royal Infirmary; Napier, Francis Horatio, St. Bartholomew's; Pagden, Trayton Charles, St. Bartholomew's; Randall, Ernest Bigwood, University College; Strugnell, Walter Thomas, St. Bartholomew's; Watson, William Ivens Buswell, Guy's; Wells, George Lee, St. Bartholomew's; William, Reginald, Muzio, St. Thomas's.

### Examination for Honours (M.B.)

Medicine.—First Class: Fleming, Percy (Scholarship and Gold Medal), University College; Tubby, Alfred Herbert (Gold Medal), Guy's; Braddon, William Leonard, Guy's; Thane, Edgar Herbert, University College; Jecks, Cyril William, University College; Smith Hugh, London Hospital; Brown, Herbert Henry, University College. Second Class: Moynihan, Berkeley Geo. Andrew, Yorkshire College; Lys, Henry Grabham, London Hospital; Deanesly, Edward, B.Sc., University College; Thompson, James Edwin, Owens College and Manchester Royal Infirmary; Featherstone, William Barltrop, Queen's College, Birmingham; Finley, Frederick Gault, McGill University, London Hospital, and Vienna; Rennie, George Edward, B.A. Sydney, University College; Stedman, Frederic Osmund, Charing Cross. Third Class: Fisher, Theodore, Guy's; Solly, Ernest, St. Thomas's; Kelsall, Henry Truman, London Hospital; Risdon, William Newt, Guy's; Wheaton, Samuel Walton, St. Thomas's; Brook, William Henry Breffit, St. Bartholomew's; Wynter, Walter Essex, Middlesex and St. Bartholomew's; Wheatley, James, King's College: Caldecott, Charles, Guy's; Godfrey, Albert Edward, Northampton Gen. Infirmary, and St. Thomas's Hospital; Luff, Arthur Pearson, B.Sc., St. Mary's; Mumford, Alfred Alexander, Owens College and Manchester Royal Infirmary; Jordan, Walter Ross, Birmingham Medical School; Taylor, Frederick Howard, London Hospital.

Obstetric Medicine.—First Class: Thane, Edgar Herbert (Scholarship and Gold Medal), University College: Jecks, Cyril William (Gold Medal), University College; Braddon, William Leonard, Guy's. Second Class: Thompson, James Edwin, Owens College and Manchester Royal Infirmary; Flemming, Percy, University College. Third Class: Brown, Herbert Henry, University College; Wynter, Walter Essex, Middlesex and St. Bartholomew's.

Forensic Medicine.—First Class: Braddon, Wm. Leonard (Scholarship and Gold Medal), Guy's; Brook, Wm. Henry Breffit (Gold Medal) St. Bartholomew's; Thompson, James Edwin, Owens College and Manchester Royal Infirmary; Deanesly, Edward, University College; Featherstone, William Barltrop, Queen's College, Birmingham; Fisher, Theodroe, Guy's. Second Class: Wheaton, Samuel Walton, St. Thomas's; Luff, Arthur Pearson, St. Mary's; Nevins, John Ernest, Liverpool School of Medicine and Guy's; Tubby, Alfred Herbert, Guy's. Third Class: Tunstall, John Ogle, University College; Brown, Herbert Henry, University College Caldecott, Charles, Guy's; Jecks, Cyril William, University College; Taylor, Frederick Howard, London Hospital; Colman, Walter Stacy; University of Edinburgh and University College.

### M.S. EXAMINATION.

Pass List.—Burghard, Frédéric Francois, Guy's; Price, Alfred Edward, M.D., Guy's; Spencer, Walter George, St. Bartholomew's; Targett, James Henry, Guy's.

### B.S. EXAMINATION.

Pass List.

First Division.—Carless, Albert, King's College; Clarke, William Frederick, Guy's; Flemming, Percy, University College; Risdon, William Newt, Guy's; Roughton, Edmund Wilkinson, M.D., St. Bartholomew's; Routh, Charles Frederic, Guy's; Tubby, Alfred Herbert, Guy's; Vincent, Herbert Edmund, Guy's; Wheatley, James, King's College; Wynter, Walter Essex, St. Bartholomew's and Middlesex.

Second Division.—Braddon, William Leonard, Guy's; Brook, William Henry Breffit, St. Bartholomew's; Brown, Herbert Henry, University College; Caldecott, Charles, Guy's; Gee, Frederick William, University College; Heatherly, Francis, Guy's; Holder, Sydney Ernest, University College; Jones, Samuel Cromwell, University College; Kelsall, Henry Truman, London Hospital; Prall, Samuel Esmond, Guy's; Stedman, Frederic Osmund, Charing Cross; Sunder, Charles Edward, University College; Taylor, Frederick Howard, London Hospital; Wells, George Lee, St. Bartholomew's.

### NOTICE TO ADVERTISERS.

The scale of charges for small prepaid Advertisements—Situations Wanted, Situations Vacant, &c., &c., is as follows:—

| | s. | d. |
|---|---|---|
| One insertion of three lines | 1 | 0 |
| Three „ „ „ | 2 | 6 |
| Per line afterwards | 0 | 6 |

*A line averages ten words.*

All copy for Advertisements must be sent to A. P. Watt, 2, Paternoster Square, E.C., by first post on the Tuesday *preceding* publication.

### NOTICE TO CORRESPONDENTS.

All correspondence must be written on one side of the paper only.

We cannot undertake to return MSS. not used.

**Communications,** whether intended for insertion or for private information, must be authenticated by the names and addresses of the writers, not necessarily for publication.

**Local papers** containing reports or news paragraphs **should be marked.**

It is especially requested that early intelligence of **local events of interest,** or which it may be thought desirable to bring under notice, may be sent direct to the Editor.

**All communications respecting editorial matters** should be addressed to the Editor, Norfolk House, Norfolk Street, Strand, London, W.C.

### Amusements with Profit for all Ages.

**Rules.**—All answers must be addressed to the Prize Editor, The Hospital, Norfolk House, Norfolk Street, Strand, W.C., not later than the *first* post on Thursday next. The full name and address must in all cases be given, but a *nom de plume* may be added for publication. Only one side of the paper must be written on.

### FOR MEN AND WOMEN.

This week credit will be given for the solution of the following:

### No. 3.—Double Acrostic.

Here read the fare the feast affords,
But do not make me eat my words.

Go, children dear, and jump about;
'Tis holiday! frisk, frolic, shout.

The cause is God's, the iron is white with heat,
See! blindfold comes th' accused with naked feet.

Who led the famous rush across the bridge of Lodi?
*On dit*, "Napoleon." Tush! 'twas I; the world's a toady.

Our craft so tight was a "ray of light,"
And our skipper's wife did the voyage write.

The schoolboy's reverence for old age
Is mostly measured by this gauge.

From dying lips, like keenest dart,
This light pierced home to Brutus' heart.

The tears were the bride's, the mistake was the man's,
They had come to be wed and forgotten the banns.

An author of maxims intended to prove
That all moral action's the fruit of self-love.

A French soldier's hat
So 'tis French—find out that.

Is this the estate? A reeking marsh of black malarial fever!
"And therefore, Tapley, now's your time to come out strong, or never."

Rejoice! but, yet remember! your years will soon be told;
And the evil days are coming when you'll be growing old.

### Answers.

No. 2. Double Acrostic.

H ac K
E v E
A c T
R igh T
T il L
H al E

Correct answers have been received from Brownie Canice, Ellice, Gretta, Pasteur, Condorcet, Reldas, Lady Clara, Dunce, Toby, Mater, Aralc, Boss, E.E., Brisbane.

### II.—Word and Literary Competition.

Commenced November 5th, 1887; ends January 28th, 1888.

Three prizes of one guinea, 15*s*. and 10*s*. 6*d*. will be given each quarter to the three competitors who obtain the highest number of marks: (1) by making the largest number of words derived from the word or phrase set for anatomical dissection; or (2) for the best answers to (6); or (3) by (1) and (2) combined.

(A) THE WORD COMPETITION.

We shall give the newspapers for dissection during this quarter, that for this week being

"The Lancet."

Observe.—Proper names, abbreviations, foreign words, words of less than four letters and repetitions are barred. Nuttall's *Standard* dictionary only to be used.

### Results of Sixth Week.

The Globe.—Ellice (212), Tim (183), Gretta (182), E. E. (179), K. M. (162), Skye (145), Boss (138), Lauriston (134), Reldas (128), Condorcet (123), Tabs (113), Kath (110), Brownie (97), Brisbane (94).

LITERARY COMPETITION.

For particulars of this see page 157, Nov. 26th.

*Gretta* sends a letter from the Cape.

### Notices to Correspondents.

*Reldas.*—No solution of No. 1 Acrostic received. Dissection of words only that week.

### THE THEATRES.

The managers of the various theatres have for the most part settled their Christmas bill, and now their irregular patrons, if we may use such a term, that is, those who only go occasionally to the play, have the hard task before them of deciding on what they had better go and see during their holidays. This is a difficulty with which the ordinary and regular playgoer has not to cope, but to others it is a question which leads to much consideration. Every year the pantomime at Drury Lane Theatre is eagerly looked forward to, and as a rule the highest anticipations are fulfilled. This year *Puss in Boots* which has been chosen is the fairy tale to afford the title rather than the subject of Mr. Augustus Harris's display at the national theatre. A Christmas without a pantomime at Drury Lane, would be something after the fashion of a *Hamlet* without the Prince. Since the withdrawal of *Pleasure*, the popularity of which certainly seemed to be falling off and which few will regret, the theatre has reechoed with preparations for the forthcoming production to be put on the stage in a still more elaborate form, if possible, than any of its predecessors. A very strong company has been engaged, and with such old favourites as Harry Nicholls, Charles Lauri and Herbert Campbell, the charming singing of Miss Wadman, and the sprightly and graceful dancing of Miss Letty Lind, Mr. Harris should be sure of suceess.

Covent Garden comes forward once more as a rival to the "house over the way," not we trust to injure the other, but offering in itself great attractions with a pantomine on *Jack and the Beanstalk*, which will merely prove a further attraction to those who like the innocent fun and pretty dances of the old-fashioned pantomime. We certainly think there is room and to spare for more than one pantomime in London.

At the Gaiety Theatre, *Miss Esmeralda*, which has proved such an unqualified success and the withdrawal of which from the evening bill must have caused many a pang to the managers, is to be succeeded by a special Christmas piece styled *Frankenstein*, wherein the old Gaiety company and the popular favourites, Miss Nelly Farren, Mr. Fred Leslie, Miss Marion Hood, and Miss Sylvia Grey will re-appear. Such, however, has been the success of *Miss Esmeralda*, that it has been decided to organise a series of matinées. As Miss Fannie Leslie goes to Covent Garden, her place as Phœbus is to be taken by Miss Jennie Rogers, and that of Miss Marion Hood by Miss Florence Dysart. Messrs. Lonnen, Stone, Thornton, etc., are to do double duty (morning and evening) and there is every prospect of the Gaiety doing a bigger business than ever before.

*Dorothy*, at the Prince of Wales' Theatre, has enjoyed an unprecedented success as one of the prettiest comic operas ever produced by English authors. *The Sultan of Mocha* at the Strand Theatre is by the same composer, but though its second act is very good, the first act is rather weak, and the third very disappointing, and it will hardly rival the popularity of Cellier's masterpiece. At the Savoy Theatre, the revival of *Pinafore* has been greeted with the greatest enthusiasm, the stage effects have been freshly worked up, and we may especially mention the manning of the yards to greet the great First Lord. It was to be feared that what with children s *Pinafores* and provincial companies the success of the piece would be problematic, but the judgment of the managers has been most fully justified.

Of other revivals the return of Mr. Toole to his pleasant little theatre with the old favourite, *The Butler*, is very welcome, and Miss Kate Phillips reappears once more as the captivating cook. We have lately mentioned other pieces and can now do no more than say that Mrs. Bernard Beere is once more drawing crowded houses by her exceedingly fine acting in that somewhat strongly-drawn play, *As in a Looking Glass.*

Mr. Wilson Barrett has taken the Globe Theatre, where he presents a new play, *The Golden Ladder*, the joint work of himself and Mr. G. R. Sims; while Mr. Terry at his new theatre in the Strand is proving a host in himself in *The Woman Hater*, and at the Criterion the *Two Roses* is extremely well done.

We have here given some plays which, with the Red Lamp and Ballad monger, both shortly to be withdrawn, the Winters Tale at the Lyceum, and the Arabian Nights in its new home at the Comedy Theatre in Panton Street, are well worth seeing, and will be sure to satisty even the most exacting sightseer.

### THE CHILDREN'S CORNER.

PRIZES.

FOR MOST CORRECT ANSWERS TO PUZZLES.

This Commenced October 1st, 1887.

One Pound a Month.

FOR BEST LITTLE LETTERS.

Ten Shillings per Quarter.

A PRIZE OF THREE GUINEAS

to the Competitor who shall have sent in the largest number of correct or best answers during the twelve months.

### Puzzles—Fourth Week.

No. 13 Historical Charade.

Beware of my *first!* 'Tis a terrible thing,
And much of disaster and woe it will bring.
My *next* is oft pinch'd and full often is squeezed
Without showing symptoms of being displeased.
It is black as a Negro, surrounded with light,
And it often is clad in a garment of white.
My *whole* was a warrior, the head of a faction,
Whose restless spirit was ever in action.

No. 14. Decapitations. — 1. Behead a European river, 'twill bring a lady's name to view. 2. I am found upon the hearth, behead me, and I become an abode which none would chose for a home.

No. 15. Enigma.

Quite round I am and have two walls
Wherein my *first* is found;
While in my *second* tender birds
Are placed when they abound.
Without my whole at Christmas-time
No festive board were crowned.

No. 16. Double Acrostic. — 1. Used in stuffing. 2. A bitter fruit. 3. A graceful flower. 4. A shellfish. 5. Gone for ever. The initials and finals are used in all decorations at Christmas-time.

### Results of Second Week Puzzles.

No. 5. Enigma.—Co-lens-o=Colenso.

No. 6. Diamond Puzzle.

S
F A R
D A N C E
S A N D O W N
C R O W S
A W L
N

No. 7. Towns in England Anagrammatically Expressed.—1. South Shields. 2. South Shields. 3. Whitehaven. 4. Leamington.

No. 8. Arithmetical Puzzle.—One boy had 5 peaches, the other had 7.

*All Right.*—A. C. K., Squeaker, Jumps, Tim, Scrooge, Pepita. *Three Right.*—Gem, Military Cadet, A. G. S., McCulloch, Holmes, Mount Edgecumbe, *Two Right.*—Spider, Snap, Judy, Plummy, Fluff, Exceisior, H. E., Fern. *One Right*—Ossian, Skye.

### Letters.

Next week write me a short account of "Canterbury Cathedral."

*Three marks each*—The Eda. *Two marks each*—Gem. *One mark each*—Fern. Ossian.

Skye, Fern, The Eda, Gem. were credited with three marks each for their letters on St. Paul's Cathedral," Ossian with two marks.

**Notice to all Correspondents.**—N.B.—All letters referring to this page, which do not arrive at Norfolk House, Norfolk Street, Strand, W.C. by the *first post on Thursdays*, and are not addressed PRIZE EDITOR, will in future be disqualified and disregarded.

No one will be permitted to win the Monthly or Quarterly Prizes twice in any one year, the annual prizes being offered especially to prevent this.

